# A Deep Learning-Augmented Density Functional Framework for Reaction Modeling with Chemical Accuracy

**DOI:** 10.1021/jacsau.5c00541

**Published:** 2025-07-24

**Authors:** Jin Xiao, Yingfeng Zhang, Bowen Li, Shuwen Zhang, Ya Gao, Wei Chen, Han Wang, John Z. H. Zhang, Tong Zhu

**Affiliations:** † Shanghai Engineering Research Center of Molecular Therapeutics and New Drug Development, School of Chemistry and Molecular Engineering, 12655 East China Normal University, Shanghai 200062, China; ‡ Shanghai Innovation Institute, Shanghai, 200003 China; § Faculty of Synthetic Biology, Shenzhen University of Advanced Technology, Shenzhen 518055, China; ∥ School of Mathematics, Physics and Statistics, Shanghai University of Engineering Science, Shanghai 201620, China; ⊥ Department of Chemistry, 66323 National University of Singapore, Singapore 117551, Singapore; # Laboratory of Computational Physics, 37580 Institute of Applied Physics and Computational Mathematics, Beijing 100088, China; ∇ NYU-ECNU Center for Computational Chemistry at NYU Shanghai, Shanghai 200062, China; ° Department of Chemistry, 447103 New York University, New York, New York 10003, United States; ◆ AI for Science Institute, Beijing 100080, China

**Keywords:** chemical reactions, DFT, machine learning, barrier height, reaction energy

## Abstract

Accurate prediction of reaction energetics remains a fundamental challenge in computational chemistry, as conventional density functional theory (DFT) often fails to reconcile high accuracy with computational efficiency. Here, we introduce Deep post-Hartree–Fock (DeePHF), a machine learning framework that integrates neural networks with quantum mechanical descriptors to achieve CCSD­(T)-level precision while retaining the efficiency of DFT to solve the reaction problems. By establishing a direct mapping between the eigenvalues of local density matrices and high-level correlation energies, DeePHF circumvents the traditional accuracy-scalability tradeoff. Trained on a limited data set of small-molecule reactions, our model demonstrates superior performance across multiple benchmark data sets, exhibiting exceptional transferability. In fact, its accuracy even surpasses that of advanced double-hybrid functionals, all while maintaining O­(N^3^) scaling. DeePHF offers a promising pathway to bridge the gap between high-level quantum chemistry methods and the practical demands for scalable, accurate models in computational chemistry, and with further refinement, it is poised to make significant contributions to the advancement of chemical reaction modeling.

## Graphical abstract

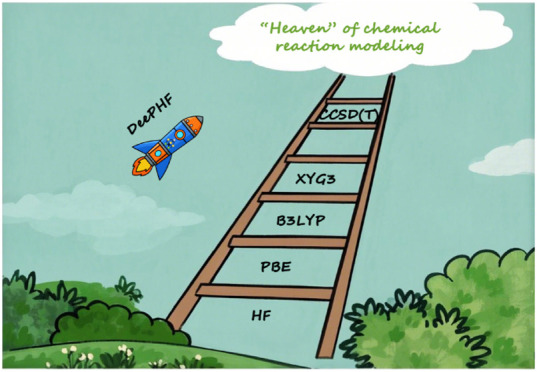

## 1 Introduction

Accurate prediction of chemical reaction energies and barrier heights is fundamental to advancing a wide range of industries, including combustion,^1–4^ catalysis,^5–9^ materials science,^10^ and pharmaceuticals.^11,12^ The activation energy (*E*_a_) dictates the reaction rate, while the reaction energy reflects thermodynamic favorability, both are fundamental to understanding reaction mechanisms at a molecular level, allowing for the design of more efficient, sustainable processes with higher yields and fewer byproducts. As industries strive for greater efficiency and sustainability, accurate reaction energy and barrier height predictions are becoming increasingly indispensable for technological innovation and the optimization of complex chemical processes.

Ideally, we hope to achieve an accuracy of 1.0 kcal/mol (or even 1.0 kJ/mol) in the calculation of these energies.^13,14^ For relatively simple gas-phase organic molecular systems (excluding cases where multireference effects need to be considered), the coupled cluster with singles and doubles and perturbative triples (CCSD­(T)) method combined with a suitable basis set can achieve this goal.^15,16^ However, the computational scaling of CCSD­(T) is proportional to the seventh power of the number of atoms, severely limiting its application to large systems or scenarios with multiple computational targets. To address this issue, many approximate methods of CCSD­(T) have been developed, such as domain-based local pair natural orbital CCSD­(T)­(DLPNO-CCSD­(T)),^17,18^ pair natural orbital CCSD­(T) (PNO-CCSD­(T)),^19^ and local natural orbital CCSD­(T) (LNO-CCSD­(T)).^20–22^ Although these methods somewhat alleviate the computational burden of the CCSD­(T) method, they do not fundamentally solve the problem.

Density functional theory (DFT) is a fundamental tool for studying chemical reactions, providing a balance between accuracy and computational efficiency.^23^ Some higher-level DFT methods, such as double-hybrid functionals, achieve accuracy that is very close to that of CCSD­(T) methods.^24^ For example, minimally empirical range-separated double-hybrid functionals, such as ωDOD60-PBEP86-D3BJ, have demonstrated exceptional performance in predicting reaction energies and barrier heights.^25^ The recently developed R-xDH7-SCC15 method effectively captures static correlation and accurately describes bond dissociation reactions.^26^ However, the computational efficiency of double-hybrid functionals is still significantly lower than commonly used DFT functionals, such as B3LYP and M06–2X. Ultimately, achieving both high accuracy and efficiency remains a challenging tradeoff. For instance, in the BH9 data set, ωB97M-V achieves a mean absolute error (MAE) of 1.26 kcal/mol for reaction energies and 1.50 kcal/mol for barrier heights.^25^ In comparison, M06–2X exhibits higher errors, with MAEs of 2.76 and 2.27 kcal/mol, respectively. Notably, B3LYP, even with the inclusion of dispersion corrections, performs significantly worse, yielding MAEs of 5.26 kcal/mol for reaction energies and 4.22 kcal/mol for barrier heights.^25–30^ The limitations of current methods highlight an urgent need for more accurate and computationally efficient approaches tailored to reaction systems.

Recent advances in machine learning (ML) have significantly enhanced the ability to model reaction energies, striking a balance between computational cost and model precision.^31–38^ Several ML models have shown great promise in predicting reaction energies and barrier heights.^35–50^ Among them, neural network potentials (NNPs) can achieve accuracy close to that of DFT while maintaining high computational efficiency. One advanced approach, delta-learning, improves upon this by not only maintaining computational efficiency but also enhancing accuracy and transferability.^51,52^ Delta-learning aims to approximate high-fidelity molecular properties by adding a statistically modeled correction to low-fidelity calculations. For instance, taking semiempirical quantum-mechanical (SQM) methods as the baseline, delta-learning can achieve or even exceed the accuracy of DFT methods. This is because SQM methods effectively enhance the physical interpretability of the model.^32,37,53,54^ Take the recently developed AIQM2 model^32,35^ as an example, it uses the DLPNO-CCSD­(T)/CBS level as the fitting target and has shown accuracy exceeding that of B3LYP-D4/6–31G*.

While delta-learning methods that incorporate SQM have made significant progress in integrating machine learning with quantum chemistry, they still face challenges in generalizing to unseen chemical spaces and achieving accuracy beyond conventional DFT. To further reduce data requirements, enhance transferability, and achieve accuracy comparable to CCSD­(T), the development of machine learning density functional theory (ML-DFT) methods has emerged. ML-DFT aims to combine the electronic structure insights inherent to DFT with the flexibility and efficiency of machine learning.^55–73^ By embedding physical principles directly into the model, ML-DFT approaches strive to achieve a better balance among accuracy, transferability, and computational cost. Cheng et al. developed the MOB-ML method, which is grounded in Nesbet’s theorem and achieves CCSD­(T)-level accuracy with strong transferability to water and small organic molecules.^56,57,74–77^ Building on this progress, Deng et al. introduced PairNet, which leverages features derived from pair natural orbitals (PNOs) to achieve high accuracy on similar data sets.^68^ Dick et al. proposed NeuralXC, a machine-learned correction to baseline density functionals, which maps electron density features to high-precision energies and forces. This approach achieves CCSD­(T)-level accuracy in predicting energies of water clusters and proton transfer barriers while maintaining the efficiency of DFT.^61^ In contrast, SPA^H^M­(a,b) focuses on encoding electronic structure information from lightweight guess Hamiltonians into local atomic/bond-density fingerprints. By combining Löwdin orthogonalization with SOAP-inspired symmetry adaptation, it outperforms traditional representations (e.g., (a)­SLATM) in predicting atomic charges and spin densities for open-shell radicals and π-conjugated dyes.^69^ In our previous work, we also proposed the Deep post-Hartree–Fock (DeePHF) and Deep Kohn–Sham (DeePKS) approaches, which generate molecular representations based on the eigenvalues of local density matrices.^62,63,78^ These methods have demonstrated significant advantages in predicting correlation energies, particularly for small drug-like molecules.^70^

Although a series of ML-DFT methods have been proposed, they primarily focus on nonreactive systems and have yet to address chemical reactions. In this work, we further develop the DeePHF method and systematically benchmark its performance in predicting reaction energies and barrier heights. The results demonstrate that DeePHF consistently achieves chemical accuracy across various reaction systems compared to the reference CCSD­(T)-F12a/cc-pVDZ-F12 level of theory,^79–81^ significantly outperforming traditional DFT and even double-hybrid functionals, which has high fidelity compared with CCSD­(T)-F12a/cc-pVTZ-F12.^2^ This article is structured as follows: Section 2 introduces the theoretical framework of DeePHF, along with data set preparation and the training procedure. Section 3 presents benchmark results and evaluates model performance. Finally, Section 4 summarizes the potential of DeePHF for accurate predictions in reaction systems.

## 2 Methods

### 2.1 Theory of the DeePHF Approach

The DeePHF approach^62,63,78^ is designed to achieve the accuracy of high-level electronic structure methods by leveraging the efficiency of low-level electronic structure calculations through machine learning, as illustrated in Figure 1.

**Figure 1:**
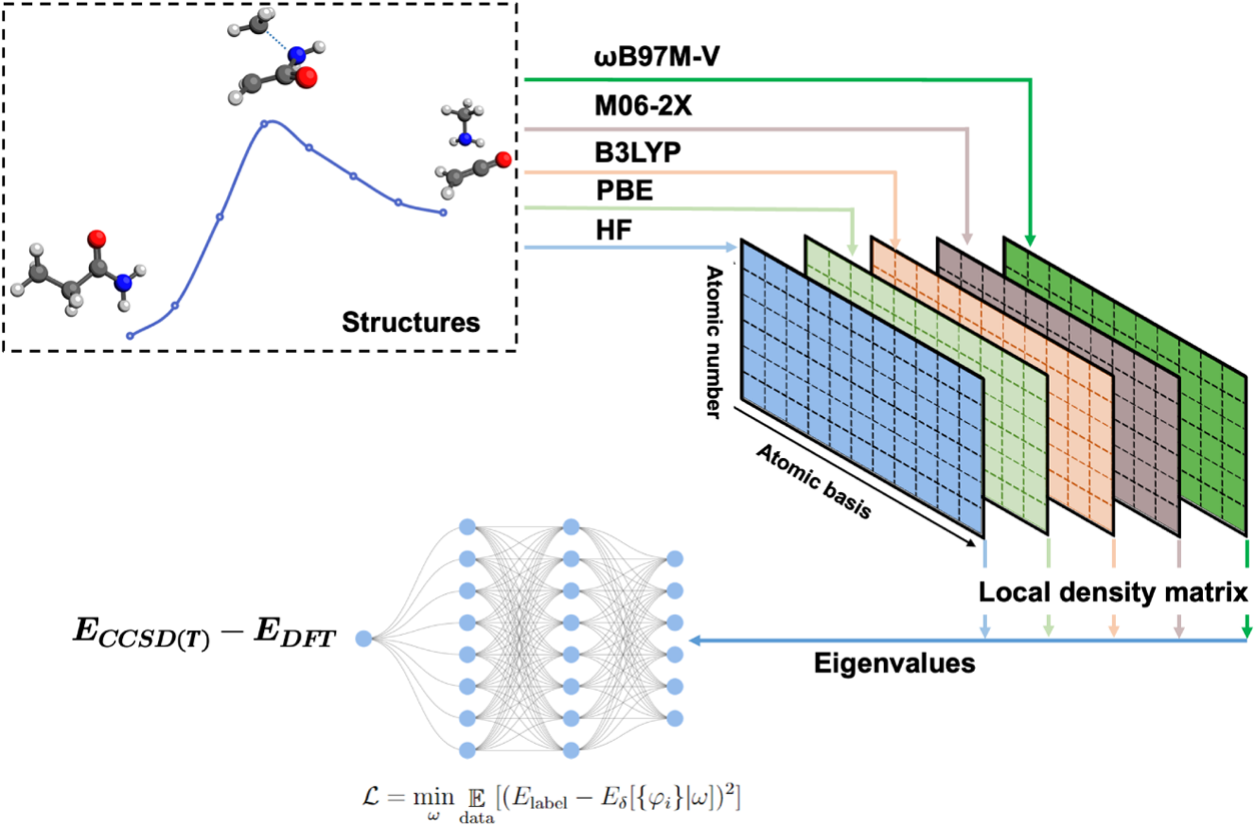
Workflow for training DeePHF models. Based on the prepared molecular structures, including reactants, products, transition states, and reaction intermediates, local electron density matrices and their eigenvalues are computed using base QM methods such as HF and PBE. These eigenvalues serve as input features for the neural network, while the target values are the energy differences between CCSD­(T)-F12 calculations and the base QM method results. After training, various DeePHF models are obtained.

The energy difference between the high-precision and low-precision methods, denoted as *E*_δ_, can be expressed as a function of the single-electron orbitals {|φ_*i*_⟩} from the low-precision method

1 $$E_{H}-E_{L}=E_{\delta }\equiv E\left[\{\left|\varphi_{i}\right\rangle \}\right]$$

If the high-precision method is CCSD­(T) and the low-precision is the Hartree–Fock­(HF) method, *E*_δ_ is the correlation energy. In the DeePHF method, a neural network is employed to establish the relationship between *E*_δ_ and the eigenvalues of the local electron density matrix. Specifically, *E*_δ_ serves as the target (label), while the eigenvalues of the local electron density matrix act as input features.

The ground-state energy of a given system is a functional of the electron density matrix Γ­(**x**, **x**′), defined as

2 $$\Gamma \left(x,x^{\prime }\right)=\sum_{i}\varphi_{i}^{*}\left(x^{\prime }\right)\varphi_{i}\left(x\right)$$

To express it in an atomic basis {α_nlm_^*I*^}, we define the local density matrix as

3 $${\left(D_{\mathrm{nl}}^{I}\right)}_{{\mathrm{mm}}^{\prime }}=\sum_{i}\left\langle \alpha_{\mathrm{nlm}}^{I}\right|\varphi_{i}\rangle \left\langle \varphi_{i}\right|\alpha_{{n\,\mathrm{lm}}^{\prime }}^{I}\rangle$$

Where *n*, *l*, *m* represent the radial, azimuthal and magnetic numbers corresponding to the atom *I*. The one-particle reduced density matrix can be obtained from various quantum chemistry methods, and it is not solely restricted to deriving it from the HF method. The eigenvalues of the local density matrix serve as input descriptors for the neural network model to ensure rotational symmetry.

4 $$d_{\mathrm{nl}}^{I}={\mathrm{EigenVals}}_{{\mathrm{mm}}^{\prime }}\left[{\left(D_{\mathrm{nl}}^{I}\right)}_{{\mathrm{mm}}^{\prime }}\right]$$

5 $$E_{\delta }=\sum_{I}F^{\mathrm{NN}}\left(d^{I}|\omega \right)$$

The loss function for training is then defined as:

6 $$L=\min_{\omega }\underset{\mathrm{data}}{E}\left[{\left(E_{\mathrm{label}}-E_{\delta }\left[\left\{\varphi_{i}\right\}|\omega \right]\right)}^{2}\right]$$

### 2.2 Data Set Preparation and Model Training

To train DeePHF models, we utilized data sets from Grambow et al.^3^ and the Transition1x data set.^82^ They only include neutral and nonradical systems along with additional data sets for the comprehensive benchmark.

#### 2.2.1 Training and Testing Set

Grambow’s data set consists of organic small-molecule reactions, including reactant, product, and transition state structures, with up to seven heavy atoms. These reactions are generated using the growing string method (GSM),^83^ and the reactants are sourced from the GDB-7 subset of GDB-17.^84^ For model training, we designate this data set as (G), which is split into training, validation, and testing sets in an 8:1:1 ratio. The test set comprises 1192 reactions and 3929 structures. To augment the training data diversity, we integrated the Transition1x data set (denoted as T). This extension of Grambow’s original data set employs Nudged Elastic Band (NEB) calculations to map 10,000 reaction pathways, producing 9.6 million geometrically distinct intermediate configurations. From it, we randomly selected 1000 reactions (8000 NEB-derived configurations) for training, 225 reactions (1800 configurations) for validation, and 287 reactions (2296 configurations) for testing For each configuration in G and T, we computed its reference energy at the CCSD­(T)-F12a/cc-pVDZ-F12 level with the Molpro software.^85^ The input features for the neural network are the eigenvalues of local electron density matrices, computed using baseline QM methods. In this work, we investigate the impact of different baseline QM methods, including HF, PBE,^86^ B3LYP,^87^ M06–2X,^88^ and ωB97M-V,^54^ on the final model performance. The PySCF software package^89,90^ was used for the feature generation.

#### 2.2.2 Additional Data Sets for Model Benchmark

To systematically evaluate model performance, we also collected several high-precision reaction data sets, including GMTKN55,^91^ BH9,^28^ and the Reaction Graph Depth 1 (RGD1) data set.^92^ GMTKN55 is widely used for benchmarking DFT models and includes seven barrier height subsets covering various reaction types such as hydrogen transfer and pericyclic reactions. We filter it to retain only reactions involving C, H, N, and O elements in neutral, closed-shell configurations. The final data set includes 84 reactions and 157 structures. BH9 is a benchmark data set of reaction barrier heights and reaction energies, constructed based on DLPNO-CCSD­(T)/CBS calculations.^28^ It consists of 449 chemical reactions spanning 9 reaction types commonly found in organic chemistry and biochemistry. After data cleaning, we retain 97 reactions and 341 molecular structures for evaluation. The RGD1 data set comprises reactions with up to 10 heavy atoms, sourced from the PubChem database.^92,93^ From this data set, we initially selected 100 reactions and optimized their structures at the ωB97X-D3/def2-TZVP level. Transition state searches were then performed, but some reactions failed to converge. As a result, we retained 83 reactions, which were subsequently relabeled at the CCSD­(T)-F12a/cc-pVDZ-F12 level for use as a test set. The final data set includes both the original and optimized geometries for evaluation. Table 1 summarizes all the test data sets used in this work. Commonly used DFTs methods ωB97X-V,^94^ ωB97X-D3,^95^ ωB97M-V,^54^ MN15,^96^ MN15-L,^97^ and M06–2X,^88^ as well as two double-hybrid functionals: XYG3 ^98,99^ and XYGJ-OS.^100,101^ were used in the benchmark.

**Table 1:** Overview of Test Datasets Used in This Work

(sub)-data set	description	method for energy calculation	method for geometry optimization	no. of reactions	no. of structures
BH76	hydrogen transfer reactions	W2-F12	unknown	4	5
BHPERI	pericyclic reactions	W1-F12	B3LYP/6–31G	22	45
		W2-F12			
BHDIV10	diverse reactions	W1-F12	PBEh-3c	4	10
		W2-F12			
INV24	inversion racemisation reactions	W1-F12	B3LYP-D3(BJ)/def2-TZVPP	13	24
		W2-F12			
		DLPNO-CCSD(T)/CBS			
BHROT27	rotation reactions	W1-F12	TPSS-D3(BJ)/def2-TZVPP	19	30
		W2-F12			
PX13	proton-exchange reactions	W1-F12	B3LYP/AVTZ	8	16
WCPT18	proton-transfer reactions	W2.2	B3LYP/AVTZ	12	19
BH9	organic reactions	DLPNO-CCSD(T)/CBS	(CAM-)B3LYP-D3/6–31(G*,+G*,+G**)	97	341
Grambow(G)	organic reactions	CCSD(T)-F12a/cc-pVDZ-F12	ωB97X-D3/def2-TZVP	1192	3929
Transition1x(T)	organic reactions	CCSD(T)-F12a/cc-pVDZ-F12	ωB97X/6–31G	287	2296
RGD1	organic reactions	CCSD(T)-F12a/cc-pVDZ-F12	B3LYP-D3/TZVP	83	249
RGD1′^a^	organic reactions	CCSD(T)-F12a/cc-pVDZ-F12	ωB97X-D3/def2-TZVP	83	249

a RGD1′ includes the same reactions as RGD1, with all structures reoptimized.

In the DeePHF model, a fully connected neural network with 3 hidden layers was used, with each layer containing 108 neurons. Before training, ridge regularization is applied. The Adam optimizer is used with a batch size of 16 and an initial learning rate of 3 × 10^–4^. The learning rate follows an exponential decay schedule with a decay factor of 0.96 every 500 epochs. Training is conducted for a total of 15,000 epochs. With this configuration, a single training run on an NVIDIA 3090 GPU takes approximately 72 h. It is important to note that the current parameter setup may not be optimal, and we will further refine the training parameters in future research.

## 3 Results and Discussion

### 3.1 Performance on Training, Validation, and Test Sets

We begin by evaluating the model’s performance in predicting absolute energies using different methods, aiming for accuracy comparable to the CCSD­(T)-F12a/cc-pVDZ-F12 reference. The DeePHF@M06–2X model, which employs M06–2X to generate baseline energies and local density matrices, achieves the optimal mean absolute error (MAE) across three data sets: G, T, and G&T (a combination of the two). Details are provided in Table 2. It can be observed that almost all the models achieve chemical accuracy. However, it must be acknowledged that they all exhibit some degree of overfitting. The performance of the DeePHF@M06–2X, DeePHF@B3LYP, and DeePHF@ωB97M-V models appear to be better than those of DeePHF@HF and DeePHF@PBE, which is understandable. After all, compared to HF and PBE, M06–2X, B3LYP, and ωB97M-V are higher-level functionals. On the one hand, they can provide more accurate features; on the other hand, since their performance is closer to that of CCSD­(T), the *E*_δ_ derived from them is smoother, making it easier to fit.

**Table 2:** Performance of DeePHF Models Using Different Base Methods across Training, Validation, and Test Sets, Reported as the Mean Absolute Error (MAE) in kcal/mol

	T			G			G&T		
data sets	train	valid	test	train	valid	test	train	valid	test
no. of structures	8000	1800	2296	32691	3845	3929	40691	5645	6225
DeePHF@HF	0.12	1.21	1.01	0.08	0.41	0.40	0.10	0.52	0.49
DeePHF@PBE	0.05	0.67	0.68	0.05	0.24	0.24	0.06	0.31	0.29
DeePHF@B3LYP	0.05	0.50	0.46	0.04	0.18	0.18	0.05	0.24	0.22
DeePHF@M06–2X	**0.05**	**0.46**	**0.39**	**0.04**	**0.18**	**0.18**	**0.05**	**0.20**	**0.20**
DeePHF@ωB97M-V	0.05	0.48	0.39	0.04	0.19	0.19	0.05	0.21	0.21

We further assess the performance of DeePHF models in predicting reaction energies and barrier heights, using test data extracted from the Grambow data set as the benchmark (labeled at the CCSD­(T)-F12a/cc-pVDZ-F12 level). In addition to the DeePHF models, we also compare the performace of several widely utilized DFT methods alongside two double-hybrid functionals. All quantum mechanical calculations were performed using the Q-Chem software package^102^ with the def2-TZVP basis set. Table 3 shows that DeePHF models outperform conventional DFTs and double-hybrid functionals in MAE and RMSE for both forward and reverse barrier heights, except for DeePHF@HF­(G) and DeePHF@HF­(G&T). DeePHF@HF exhibits slightly higher RMSE than XYGJ-OS for reverse barrier heights (1.76 and 1.80 kcal/mol in the G and G&T data sets, respectively). Most conventional DFT methods perform poorly in predicting barrier heights, while ωB97M-V, showing better accuracy in reaction energy predictions among them. Double-hybrid functionals achieve significantly higher precision than standard DFT methods, yet they still fall short compared to DeePHF models. Among them, DeePHF@B3LYP­(G&T) achieves the lowest RMSE for barrier heights, with 0.67 kcal/mol for forward reactions and 0.71 kcal/mol for reverse reactions, while DeePHF@M06–2X­(G&T) attains the best RMSE for reaction energies at 0.22 kcal/mol. For comparison, the base functional of DeePHF@M06–2X­(G&T), M06–2X, has significantly higher RMSE values of 5.35 kcal/mol (forward) and 5.17 kcal/mol (reverse) for barrier heights, and 2.68 kcal/mol for reaction energy. Although ωB97M-V performs better than M06–2X, DeePHF@ωB97M-V­(G&T) achieves accuracy comparable to DeePHF@M06–2X­(G&T). Compared to other machine learning models trained on Grambow’s data sets^39–41^ for predicting reaction barriers, the DeePHF method, which relies on descriptors derived from quantum chemical calculations, is indeed less efficient. However, it achieves obviously higher accuracy. Moreover, the primary objective of the DeePHF method is not limited to predicting activation energies. It is also capable of accurately computing the energies of diverse configurations along reaction pathways. Although its computational cost is comparable to that of DFT, its accuracy approaches CCSD­(T) level, making it well-suited for efficiently generating high-quality labeled data to support the development of machine learning-based potential energy surfaces.

**Table 3:** Accuracy of DeePHF Models and DFT Methods was Benchmarked against CCSD­(T)-F12a/cc-pVDZ-F12 Reference Data Based on the Grambow Dataset, Evaluating Both Barrier Heights (BH) and Reaction Energies (RE)^a^

	BH (forward)		BH (reverse)		RE	
error (kcal/mol)	MAE	RMSE	MAE	RMSE	MAE	RMSE
ωB97X-V	4.25	6.31	3.24	5.37	2.63	3.41
MN15-L	3.94	4.77	2.96	3.86	3.26	4.24
ωB97X-D3	3.71	5.58	3.02	4.89	2.27	2.88
M06–2X	3.37	5.35	3.26	5.17	2.10	2.68
MN15	2.53	3.83	2.78	3.95	2.49	3.22
ωB97M-V	2.60	4.50	2.59	4.38	1.44	1.92
XYG3	1.52	2.00	1.43	1.91	1.39	1.77
XYGJ-OS	1.43	1.81	1.36	1.75	1.13	1.46
DeePHF@HF(G)	0.97	1.67	1.04	1.76	0.33	0.69
DeePHF@PBE(G)	0.54	0.89	0.59	0.95	0.20	0.43
DeePHF@B3LYP(G)	0.42	0.76	0.45	0.79	0.14	0.26
DeePHF@M06–2X(G)	0.43	0.83	0.47	0.87	0.14	0.27
DeePHF@ωB97M-V(G)	0.46	0.92	0.48	0.95	0.14	0.31
DeePHF@HF(G&T)	0.95	1.75	1.02	1.80	0.29	0.57
DeePHF@PBE(G&T)	0.52	0.87	0.65	1.05	0.21	0.38
DeePHF@B3LYP(G&T)	**0.39**	**0.67**	**0.43**	**0.71**	0.15	0.26
DeePHF@M06–2X(G&T)	0.41	0.84	0.43	0.86	**0.12**	**0.22**
DeePHF@ωB97M-V(G&T)	0.42	0.90	0.45	0.94	0.13	0.29

a Barrier height analysis was performed with explicit separation of forward and reverse reaction directions. All errors are reported as MAE and RMSE in kcal/mol, with optimal values emphasized in bold

Although DeePHF models demonstrate satisfactory performance in predicting barrier heights in Grambow’s data sets, we further assessed their capability in modeling energies of nonequilibrium reaction intermediates. From the Transition1x data set, 287 reactions were selected, each containing 8 structures along the minimum energy path (MEP) including reactant and product states. All structures were re-evaluated at the CCSD­(T)-F12a/cc-pVDZ-F12 level to establish reference energies. Figure 2(a) displays the MEP profile of a representative reaction, where DeePHF@M06–2X­(G&T) shows remarkable agreement with CCSD­(T)-F12a results. We computed relative energies (versus reactants) for all 287 paths using various methods and evaluated their deviations from CCSD­(T)-F12a references, as summarized in Figure 2(b). Among conventional DFT methods, ωB97M-V demonstrates the best performance with an error approaching 1 kcal/mol. Notably, XYGJ-OS achieves an impressive error below 1 kcal/mol. Except for DeePHF@HF­(T), all DeePHF models maintain errors under 1 kcal/mol, with DeePHF@M06–2X­(G&T) achieving the optimal MAE of 0.27 kcal/mol. DeePHF@B3LYP­(G&T) and DeePHF@ωB97M-V­(G&T) show comparable accuracy. Importantly, the expanded training set (G&T) consistently outperforms the smaller set (T), which aligns with expectations. To further increase the challenge, we tasked these models with predicting relative energies of two structures adjacent to the transition state (TS) versus the TS itself. Results are shown in Figure 2(b). DeePHF@M06–2X­(G&T) again demonstrates superior performance with an MAE of 0.52 kcal/mol. However, all models exhibit increased errors for this more demanding task. XYGJ-OS emerges as the top-performing functional with an MAE around 1 kcal/mol, while MN15, the best conventional DFT model, approaches 2 kcal/mol. Notably, the majority of DeePHF models maintain errors below 1 kcal/mol, highlighting their robustness. More details can be found in Tables S1 and S2.

**Figure 2:**
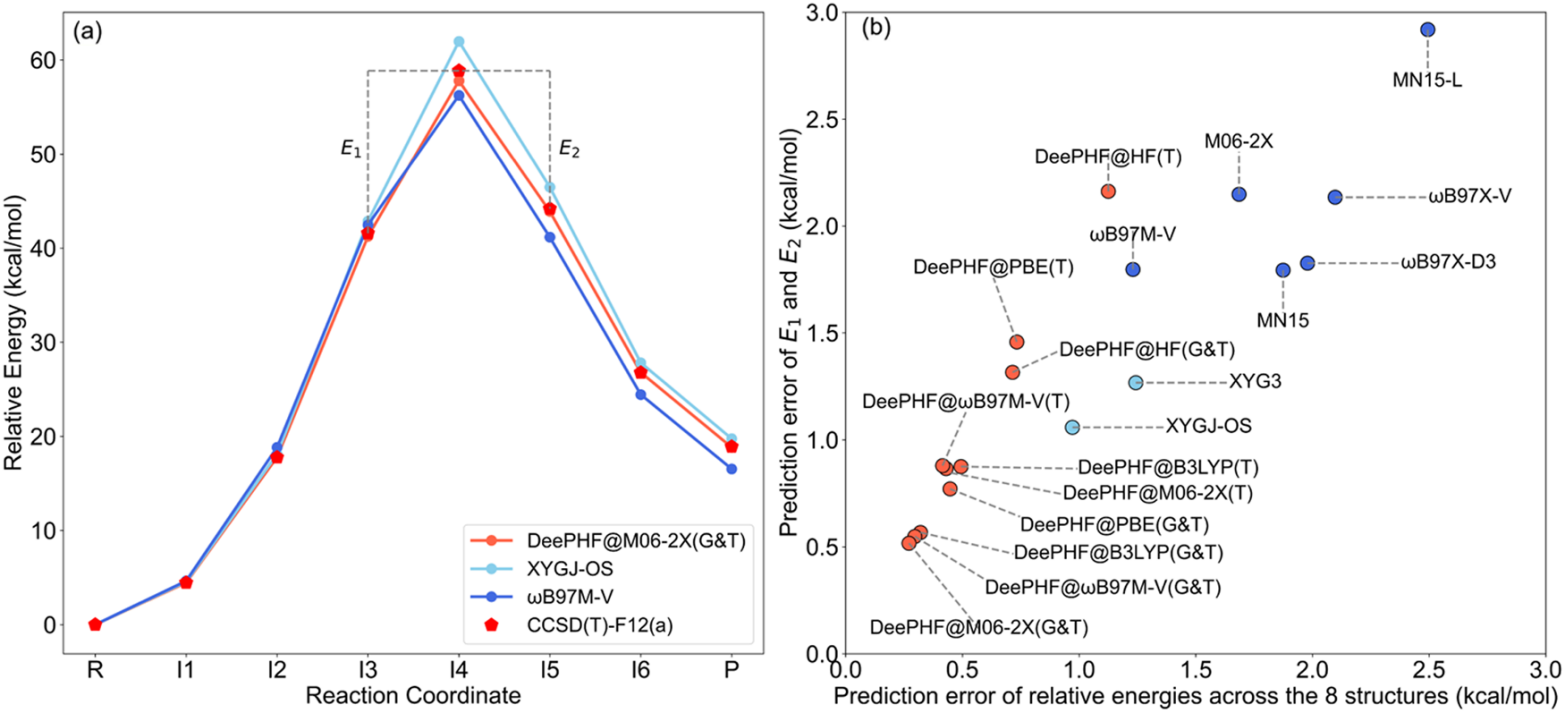
Performance of DeePHF models on nonequilibrium structures. (a) Minimum energy path (MEP) of a randomly selected reaction from the Transition1x test set (There are totally 287 reactions, and the MEP of each reaction is defined by 8 structures along the path). With CCSD­(T)-F12a as the reference, the performance of DeePHF@M06–2X­(G&T), XYGJ-OS, and ωB97M-V is compared. (b) MAE results for different methods in predicting relative energies across 8 conformations per reaction in the Transition1x test set and the relative energies of two adjacent structures on either side of the TS, denoted as E_1_ and E_2_ in (a). Orange points represent DeePHF models, blue ones denote conventional DFT methods, and sky-blue points indicate double-hybrid functionals.

### 3.2 Performance on the Independent Test Sets RGD1 and RGD1′

The RGD1 database^92,103,104^ contains approximately 170,000 reactions with molecules up to 10 heavy atoms. Each reaction includes reactant, product, and transition state structures optimized at the B3LYP-D3/TZVP level. From this data set, we randomly selected 100 reactions for independent model evaluation. After intrinsic reaction coordinate (IRC) confirmation and reoptimization in ωB97X-D3/def2-TZVP, 83 reactions successfully converged to valid TS structures. These converged reactions, with both original and optimized geometries, were subsequently evaluated at the CCSD­(T)-F12a/cc-PVDZ-F12 level and divided into two data sets based on the optimization method­(referred to as RGD1 and RGD1’ in this work).

As shown in Figure 3, DeePHF@B3LYP­(G&T) achieved the best performance for barrier heights using the original geometries (optimized at the B3LYP-D3/TZVP level), with an MAE of 1.88 kcal/mol. For reaction energy predictions, DeePHF@M06–2X­(G&T) outperformed all other models, achieving an MAE of 0.43 kcal/mol, demonstrating the strong transferability of DeePHF models for stable molecular structures. After reoptimizing the geometries at the ωB97X-D3/def2-TZVP level, the overall performance of DeePHF models improved, particularly for barrier heights. DeePHF@ωB97M-V­(G&T) achieved the optimal MAE of 1.13 kcal/mol, approaching chemical accuracy. DeePHF@M06–2X­(G&T) and DeePHF@B3LYP­(G&T) both followed closely, with an MAE of 1.20 kcal/mol.

**Figure 3:**
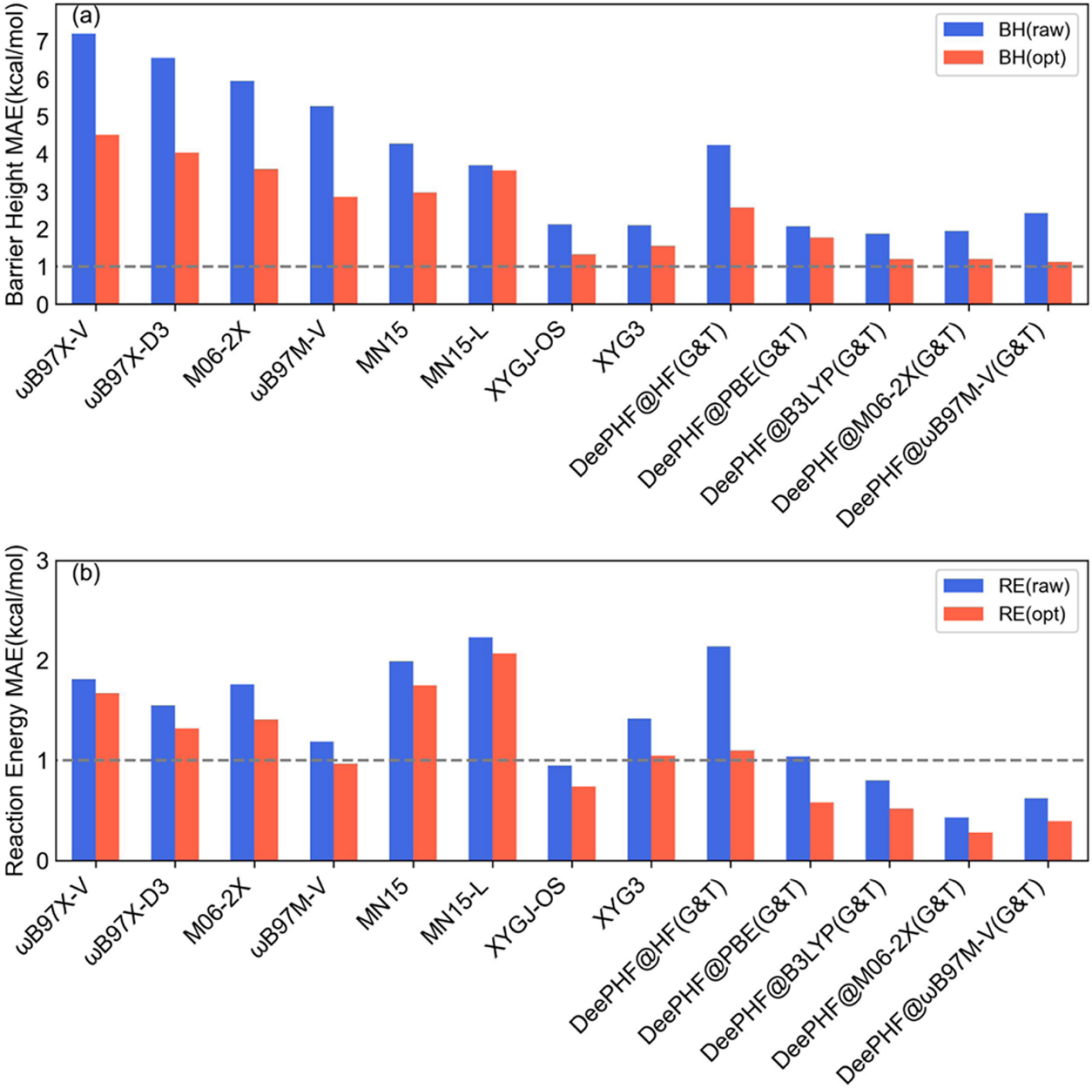
Model performance was evaluated on independent test sets. From the RGD1 database, 83 reactions (including reactants, products, and transition state structures) were randomly selected. These structures were initially optimized at the B3LYP-D3/TZVP level. Subsequently, these structures have undergone intrinsic reaction coordinate (IRC) confirmation and optimization at the ωB97X-D3/def2-TZVP level to form the RGD1’ data set. Reference energies for all structures in both data sets were recalculated at the CCSD­(T)-F12a/cc-PVDZ-F12 level. (a) MAE of barrier height, (b) MAE of reaction energy. The blue and orange boxs represent the RGD1 and RGD1’ data sets, respectively.

When tested on the original geometries, DeePHF@ωB97M-V­(G&T) exhibited slightly lower accuracy in barrier height predictions than DeePHF@PBE­(G&T). Similarly, DeePHF@HF­(G&T) performed worse than MN15 and MN15-L. However, after adopting the reoptimized structures, DeePHF@M06–2X­(G&T) and DeePHF@ωB97M-V­(G&T) surpassed DeePHF@PBE­(G&T) in barrier height prediction accuracy, and DeePHF@HF­(G&T) outperformed MN15 and MN15-L, indicating that optimized transition-state structures have a significant impact on barrier height predictions.

Despite these improvements, conventional DFTs and double-hybrid functionals continued to show relatively poor accuracy in predicting barrier heights. The ωB97M-V functional, for example, yielded an MAE of 2.86 kcal/mol, while XYGJ-OS performed better but still exhibited an MAE of 1.32 kcal/mol. While double hybrids improve upon standard DFT methods, they still lag behind DeePHF models, particularly after geometry optimization. The superior accuracy of DeePHF models over conventional DFT and double-hybrid functionals underscores the advantages of the ML-DFT framework. The improvements observed after geometry optimization further emphasize the importance of accurate transition-state structures for precise barrier height predictions. Moreover, DeePHF models exhibit strong transferability. Despite being trained on the G&T data sets, which primarily consist of small organic molecules, they maintain high accuracy on the RGD1 data set, which includes a more diverse range of reactions.

For a detailed comparison, Tables S3 and S4 present the MAE and RMSE values for forward and reverse barrier heights across different methods.

### 3.3 Performance on the BH9 Data Set

The BH9 data set, which consists of various organic reactions, provides an opportunity to test model performance on more complex molecules. After filtering, the data set includes molecules with up to 31 heavy atoms. Figure 4 reveals a positive correlation between predicted reaction energies and barrier heights. Among all models, DeePHF@M06–2X­(G&T) achieves the best performance, with MAEs of 1.08 kcal/mol for barrier heights and 0.70 kcal/mol for reaction energies, closely followed by DeePHF@ωB97M-V­(G&T). However, DeePHF@B3LYP­(G&T) performs slightly worse, with MAEs of 1.62 kcal/mol for reaction energies and 1.90 kcal/mol for barrier heights. Among other methods, ωB97M-V, ωB97X-D3, DeePHF@B3LYP­(G&T), and DeePHF@PBE­(G&T) yield similar MAEs for reaction energies, ranging from 1.60 to 1.75 kcal/mol, but their barrier height predictions vary significantly, from 1.51 kcal/mol for ωB97M-V to 3.24 kcal/mol for ωB97X-D3. The double-hybrid functional XYGJ-OS ranks as the third-best method overall, with MAEs of 1.34 kcal/mol for barrier heights and 1.32 kcal/mol for reaction energies, while XYG3 performs slightly worse, with MAEs of 2.88 and 2.05 kcal/mol, respectively.

**Figure 4:**
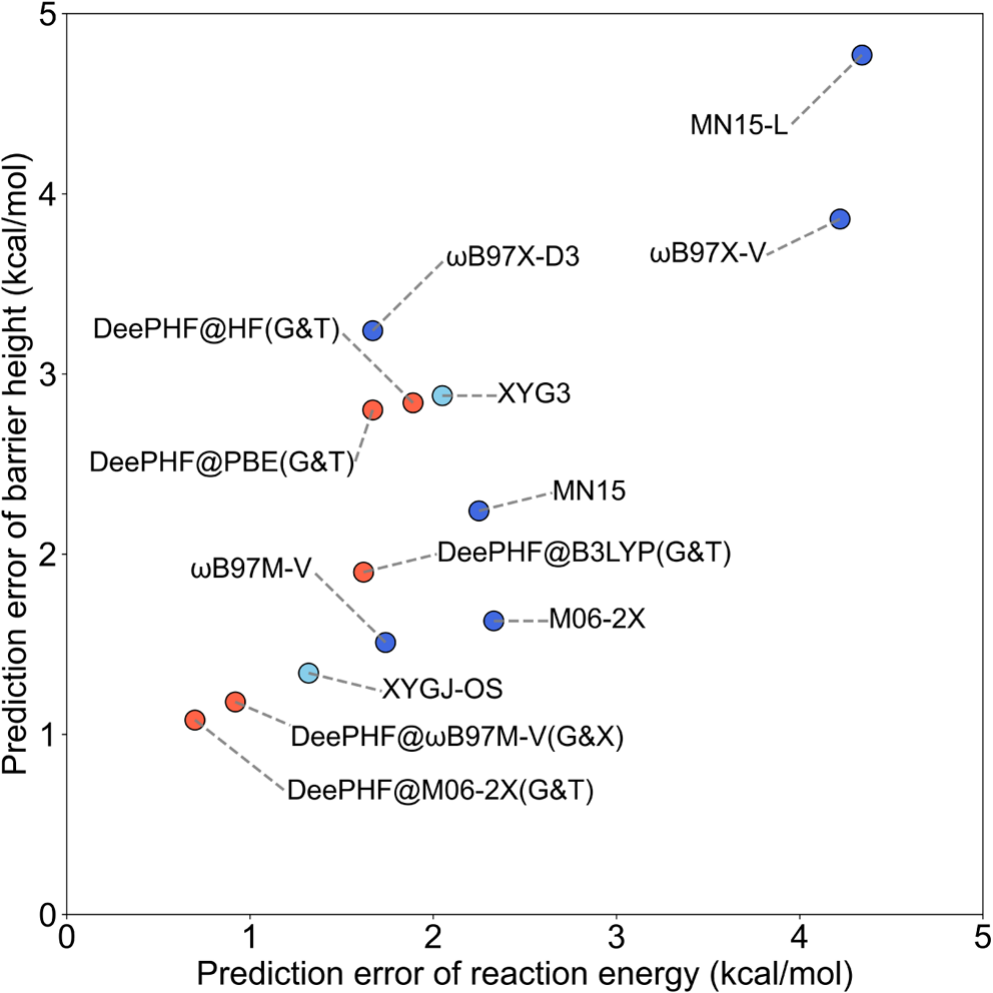
Model performance on barrier heights and reaction energies in BH9 data set. MAE for reaction energies and barrier heights are plotted for various methods. Purple points represent DeePHF models, blue points denote conventional DFT methods, and sky-blue points indicate double-hybrid functionals. The performance of DeePHF models is consistently superior.

After excluding reactions involving elements other than C, H, O, and N, the BH9 data set contains 97 reactions across six reaction types: Diels–Alder reactions, cyclo-addition, electrocyclic reactions, intramolecular reactions, rearrangement reactions, and proton transfer.^70^ DeePHF@M06–2X­(G&T) generally maintains chemical accuracy across these categories but exhibits slightly higher errors in Diels–Alder reactions, with a barrier height MAE of 1.41 kcal/mol (Table S5). Notably, in cyclo-addition reactions, DeePHF@M06–2X­(G&T) is the only model that achieves an MAE close to 1 kcal/mol, with 0.74 kcal/mol for reaction energies and 1.01 kcal/mol for barrier heights. Furthermore, we evaluated the computational efficiency of the DeePHF models. Specifically, for the BH9 reaction index 07–1 (containing 12 atoms), DeePHF@M06–2X required 28.4 min to calculate the single-point energies of the reactant, product, and transition state, whereas CCSD­(T)-F12a required more than 200 min on a single Intel­(R) Xeon­(R) Gold 6129 CPU.

### 3.4 Performance on the GMTKN55 Barrier Height Data Set

At the final stage of evaluation, we assessed model performance on barrier height subsets of the GMTKN55 database. As shown in Table 4, across the complete set of 84 reactions, XYG3 achieved the best MAE of 0.80 kcal/mol, followed by DeePHF@ωB97M-V­(G&T) and DeePHF@M06–2X­(G&T) with MAEs of 1.31 and 1.33 kcal/mol, respectively. It should be noted that some subsets of GMTKN55 were used in the development of the DFT models adopted in this study, whereas the DeePHF models were trained entirely without using this data set. The DeePHF models exhibited higher errors on the PX13 and INV24 subsets. The PX13 subset consists of proton-exchange reactions involving multimeric structures such as trimers and hexamers, which are absent from the training data set. The INV24 subset presents a different challenge, primarily comprising inversion and racemization reactions among macrocyclic aromatic hydrocarbons, another reaction type not represented in the training data. The lack of training coverage for these specialized reactions likely contributes to the reduced performance of DeePHF models on these subsets.

**Table 4:** Performance of DeePHF Models and DFT Methods on Seven Barrier Height Subsets from GMTKN55, Reported as MAE (kcal/mol)^a^ ^b^

data sets	BH76	BHPERI	BHDIV10	BHROT27	WCPT18	PX13	INV24	total^1^	total^2*^
no. of reactions	5	22	5	19	12	8	13	84	63
MN15-L	2.35	1.91	2.79	0.84	1.78	4.66	1.05	1.86	1.50
M06–2X	1.03	1.60	1.11	0.39	2.51	6.21	1.27	1.78	1.65
ωB97X-D3	0.88	2.65	1.04	0.37	2.45	2.60	1.16	1.67	1.34
MN15	1.92	1.43	1.11	0.46	2.45	2.59	2.30	1.61	1.67
ωB97X-V	1.01	1.99	1.31	0.29	2.78	3.36	1.06	1.61	1.32
ωB97M-V	1.55	1.17	1.67	0.25	2.90	3.20	1.17	1.46	1.29
XYGJ-OS	1.39	2.17	1.54	0.32	1.79	2.65	0.72	1.43	1.35
XYG3	0.68	**0.49**	1.68	0.34	1.66	**1.45**	**0.54**	**0.80**	1.43
DeePHF@HF(G&T)	2.79	1.65	1.41	0.56	1.38	6.89	2.27	2.01	1.05
DeePHF@PBE(G&T)	1.10	1.07	0.77	0.43	2.08	6.82	2.74	1.86	0.78
DeePHF@B3LYP(G&T)	0.65	0.61	**0.57**	0.29	1.36	6.76	1.68	1.40	0.66
DeePHF@M06–2X(G&T)	0.69	0.60	0.81	0.20	**0.98**	4.83	2.86	1.33	**0.58**
DeePHF@ωB97M-V(G&T)	**0.31**	0.74	0.71	**0.15**	1.22	4.64	2.62	1.31	0.62

a The best results are highlighted in bold.

b Total^2^* remove the PX13 and INV24 subsets.

After excluding PX13 and INV24, the overall performance of DeePHF models improved significantly. DeePHF@M06–2X­(G&T) achieved the best MAE of 0.58 kcal/mol, demonstrating its superior accuracy on the remaining 63 reactions. Among conventional DFT methods, ωB97M-V performed the best, with an MAE of 1.29 kcal/mol, outperforming both XYGJ-OS and XYG3. These results demonstrate that DeePHF models have the potential to achieve chemical accuracy in a wide range of similar systems, even when trained with limited data. However, Table 4 also emphasizes the necessity of incorporating additional small amounts of training data for specialized reaction types (such as those found in PX13 and INV24) in future studies, in order to further enhance the model’s robustness and generalization ability. We also compared the performance on other subsets of GMTKN55, including reaction energies for small systems, reaction energies for large systems, isomerization reactions, and both intermolecular and intramolecular noncovalent interactions. The detailed results are summarized in Table S6. Across the selected subsets, DeePHF@M06–2X consistently achieves the highest overall accuracy. However, its performance is slightly inferior to conventional DFT methods for intermolecular and intramolecular noncovalent interactions, likely because such systems were not included in its training data.

## 4 Conclusions

In this work, we present a machine learning-enabled density functional framework (DeePHF) that maintains superior accuracy in modeling chemical reaction energetics while ensuring computational efficiency comparable to conventional DFT. By integrating deep neural networks with quantum mechanical descriptors, our approach effectively bridges the accuracy gap between low-cost DFT functionals and high-level wave function theories. Three key advancements emerge from this study:

First, the DeePHF method demonstrates a remarkable synergy between data-driven modeling and electronic structure theory. When benchmarked against CCSD­(T)-F12a references across diverse reaction data sets, DeePHF@M06–2X achieves mean absolute errors of 0.70–1.08 kcal/mol for reaction energies and barrier heights—surpassing even advanced double-hybrid functionals like XYGJ-OS (1.32–1.34 kcal/mol) while retaining the O­(N^3^) scaling characteristic of its base functional. Given its robustness across the benchmark sets, DeePHF@M06–2X is highly recommended for applications. Second, the method’s architecture exhibits exceptional transferability. The eigenvalue-based representation of local density matrices enables robust predictions for larger molecules and nonequilibrium configurations along reaction paths. Third, DeePHF can be used for generating machine-learned potential energy surfaces. The demonstrated capability to predict CCSD­(T)-level energies from DFT-grade computations positions it as a versatile tool for constructing high-fidelity training data sets in ML-potential development, particularly for complex reaction networks where direct CCSD­(T) sampling remains prohibitive.

Despite these promising results, there remain several avenues for future improvement. First, while DeePHF models achieve high accuracy in energy predictions, the rapid computation of first- and second-order derivatives continues to be an area requiring further investigation. Although the DeePHF framework is fully capable of training on gradients, obtaining CCSD­(T)-level gradients remains extremely challenging. In future work, we will explore potential solutions to this issue. Additionally, the model’s performance in more complex and diverse reaction types should be further improved by expanding the training data sets to include more challenging reaction types, such as those involving charged organic molecules, radicals, and organometallic systems. Lastly, the computational efficiency of DeePHF models can be further optimized by developing faster base methods.

In conclusion, DeePHF provides a path forward for bridging the gap between high-level quantum chemistry methods and the practical need for scalable, accurate models in computational chemistry. With further refinement, DeePHF stands to contribute significantly to the advancement of chemical reaction modeling, particularly in industries focused on sustainable energy, catalysis, and drug development.

## Supplementary Material



## Data Availability

The data are available on Zenodo at 10.5281/zenodo.14876882. The source codes and the models are available via https://github.com/Eipgen/BHpredict.

## Notes

Supporting Information provides additional benchmark results for DeePHF models in reaction modeling; Tables S1 and S2 evaluate relative energies in the Transition1x data set (287 reactions), including both equilibrium and nonequilibrium structures; Tables S3 and S4 assess model performance on RGD1 (83 reactions), comparing original and optimized geometries; Table S5 examines barrier heights and reaction energies in BH9 (97 reactions, 6 reaction types); Table S6 gives the performance in the selected subsets of GMTKN55­(29 subsets, 643 reactions) (PDF)
