# Supplementary material for: A Deep Learning-Augmented Density Functional Framework for Reaction Modeling with Chemical Accuracy
Source: JACS Au. 2025 Jul 24;5(8):3892–903. doi: 10.1021/jacsau.5c00541 (PMC12381711; doi:10.1021/jacsau.5c00541)
Supplement: Supplementary file 1 [file au5c00541_si_001.pdf]

Supporting Information

A Deep Learning-Augmented Density Functional Framework for Reaction Modeling with Chemical Accuracy

Jin Xiao[a,b], Yingfeng Zhang[c], Bowen Li[a], Shuwen Zhang[a], Ya Gao[d], Wei Chen[e], Han Wang[f], John Z.H. Zhang[*,c,g,h,], and Tong Zhu[*,a,g,i,]

[a]Shanghai Engineering Research Center of Molecular Therapeutics and New Drug Development, School of Chemistry and Molecular Engineering, East China Normal University at Shanghai, 200062, China
[b]Shanghai Innovation Institute, Shanghai, 200003 China
[c]Faculty of Synthetic Biology, Shenzhen University of Advanced Technology, Shenzhen 518055, China
[d]School of Mathematics, Physics and Statistics, Shanghai University of Engineering Science, Shanghai 201620, China
[e]Department of Chemistry, National University of Singapore, Singapore 117551, Singapore
[f]Laboratory of Computational Physics, Institute of Applied Physics and Computational Mathematics, Beijing 100088, China
[g]NYU-ECNU Center for Computational Chemistry at NYU Shanghai, Shanghai 200062, China
[h]Department of Chemistry, New York University, NY, NY 10003, USA
[i]AI for Science Institute, Beijing 100080, P.R. China

john.zhang@nyu.edu; tongzhu.work@gmail.com

Table S1: Root mean square error (RMSE) and mean absolute error (MAE) of relative energies for 287 reactions extracted from the Transition1x dataset. Each reaction includes 8 structures, representing the reactant, product, and transition state. Errors are reported in kcal/mol, with CCSD(T)-F12a/cc-PVDZ-F12 as the reference level. The best results are highlighted in bold.

	RMSE	MAE
MN15-L	3.72	2.49
ωB97X-V	3.22	2.10
ωB97X-D3	2.97	1.98
MN15	2.69	1.87
M06-2X	2.71	1.68
XYG3	1.75	1.24
ωB97M-V	2.10	1.23
DeePHF@HF(T)	1.85	1.13
XYGJ-OS	1.31	0.97
DeePHF@PBE(T)	1.22	0.73
DeePHF@HF(G&T)	1.14	0.72
DeePHF@B3LYP(T)	0.79	0.49
DeePHF@PBE(G&T)	0.69	0.45
DeePHF@M06-2X(T)	0.76	0.43
DeePHF@ωB97M-V(T)	0.75	0.42
DeePHF@B3LYP(G&T)	0.51	0.32
DeePHF@ωB97M-V(G&T)	0.54	0.30
DeePHF@M06-2X(G&T)	**0.45**	**0.27**

Table S2: RMSE and MAE of relative energies for two adjacent non-equilibrium structures on either side of the TS in the Transition1x dataset (287 reactions). Errors are reported in kcal/mol, with CCSD(T)-F12a/cc-PVDZ-F12 as the reference level. The best results are highlighted in bold.

	RMSE	MAE
MN15-L	3.86	2.92
DeePHF@HF(T)	3.26	2.16
M06-2X	3.39	2.15
ωB97X-V	3.41	2.13
ωB97X-D3	2.97	1.83
ωB97M-V	2.92	1.80
MN15	2.63	1.79
DeePHF@PBE(T)	2.07	1.46
DeePHF@HF(G&T)	1.92	1.32
XYG3	1.72	1.27
XYGJ-OS	1.44	1.06
DeePHF@ωB97M-V(T)	1.44	0.88
DeePHF@B3LYP(T)	1.34	0.88
DeePHF@M06-2X(T)	1.33	0.87
DeePHF@PBE(G&T)	1.06	0.77
DeePHF@B3LYP(G&T)	0.83	0.57
DeePHF@ωB97M-V(G&T)	0.97	0.55
DeePHF@M06-2X(G&T)	**0.81**	**0.52**

Table S3: RMSE and MAE of models on RGD1 (83 reactions) using original geometries. Errors are reported in kcal/mol, with CCSD(T)-F12a/cc-PVDZ-F12 as the reference level. The best results are highlighted in bold.

	BH(Forward)		BH(Reverse)		RE	
**Error(kcal/mol)**	**MAE**	**RMSE**	**MAE**	**RMSE**	**MAE**	**RMSE**
ωB97X-V	7.40	11.04	7.02	10.86	1.81	2.43
ωB97X-D3	6.72	9.82	6.39	9.62	1.55	2.08
M06-2X	6.25	9.03	5.65	8.81	1.76	2.53
MN15	4.45	6.83	4.09	6.64	1.99	2.70
MN15-L	3.54	4.16	3.85	4.68	2.23	2.99
ωB97M-V	5.45	8.73	5.10	8.62	1.19	1.74
XYG3	2.18	3.30	2.01	3.04	1.42	2.15
XYGJ-OS	2.20	3.62	2.05	3.35	0.95	1.75
DeePHF@HF(G&T)	4.48	6.73	3.99	5.95	2.14	3.81
DeePHF@PBE(G&T)	2.09	2.84	2.05	2.78	1.04	1.51
DeePHF@B3LYP(G&T)	**1.87**	**2.50**	**1.89**	**2.64**	0.80	1.14
DeePHF@M06-2X(G&T)	1.94	3.19	1.96	3.10	**0.43**	**0.69**
DeePHF@ωB97M-V(G&T)	2.49	4.35	2.37	4.29	0.62	1.02

Table S4: RMSE and MAE of models on RGD1' (83 reactions) using optimized geometries. Errors are reported in kcal/mol, with CCSD(T)-F12a/cc-PVDZ-F12 as the reference level. The best results are highlighted in bold.

	BH(Forward)		BH(Reverse)		RE	
**Error(kcal/mol)**	**MAE**	**RMSE**	**MAE**	**RMSE**	**MAE**	**RMSE**
ωB97X-V	4.52	6.66	4.51	6.60	1.67	2.24
ωB97X-D3	4.09	5.95	3.98	5.87	1.32	1.76
M06-2X	3.71	5.52	3.48	5.51	1.41	1.95
ωB97M-V	3.30	4.23	3.39	4.08	2.57	3.33
MN15	3.27	3.97	3.86	4.63	2.07	2.79
MN15-L	2.91	4.82	2.80	4.79	0.97	1.36
XYG3	1.59	2.23	1.50	2.11	1.05	1.39
XYGJ-OS	1.32	2.01	1.33	2.00	0.74	1.03
DeePHF@HF(G&T)	2.65	3.43	2.50	3.33	1.10	1.61
DeePHF@PBE(G&T)	1.77	2.30	1.80	2.35	0.58	0.80
DeePHF@B3LYP(G&T)	1.18	**1.61**	1.22	1.68	0.52	0.73
DeePHF@M06-2X(G&T)	1.21	1.77	1.19	1.76	**0.28**	**0.39**
DeePHF@ωB97M-V(G&T)	**1.16**	1.75	**1.09**	**1.68**	0.39	0.52

Table S5: MAEs of barrier heights and reaction energies across different reaction types in the BH9 dataset, with errors reported in kcal/mol. The reference energy is DLPNO-CCSD(T)/CBS. The best results are highlighted in bold.

Reaction types	Diels-Alder		Cycloaddition		Electrocyclic		Intramolecular		Rarrangement		Proton transfer	
No. of reactions	35		21		15		9		14		3	
	BH	RE	BH	RE	BH	RE	BH	RE	BH	RE	BH	RE
MN15-L	4.58	3.66	6.85	7.06	1.40	1.70	8.19	7.15	4.17	3.82	1.71	0.40
ωB97X-D3	3.28	1.90	3.17	1.36	2.28	1.51	4.35	1.60	3.98	1.81	1.17	1.46
ωB97X-V	3.79	4.88	3.95	5.62	2.82	2.89	5.84	6.03	4.31	1.39	1.30	1.00
XYG3	2.69	1.68	4.39	5.09	1.05	0.44	4.47	1.85	2.40	**0.51**	0.98	0.97
MN15	2.27	1.98	3.57	3.23	0.53	0.85	2.82	2.49	1.72	3.12	2.06	0.60
XYGJ-OS	**1.22**	1.01	1.68	3.35	1.46	**0.22**	**0.92**	**0.69**	1.28	0.52	1.38	1.84
M06-2X	1.78	2.75	2.43	3.30	0.75	0.64	1.51	2.74	1.10	1.81	1.43	0.30
ωB97M-V	1.33	1.76	1.64	2.71	0.98	0.79	2.47	2.31	1.72	1.23	1.44	0.21
DeePHF@HF(G&T)	2.95	2.29	3.41	2.51	2.49	0.47	3.06	3.26	2.42	0.99	0.58	0.21
DeePHF@PBE(G&T)	2.69	1.68	2.55	3.50	2.09	0.98	6.05	0.74	2.38	0.60	1.79	0.08
DeePHF@B3LYP(G&T)	1.91	1.76	1.89	2.76	1.57	0.49	2.54	0.98	2.03	1.46	0.93	0.17
DeePHF@M06-2X(G&T)	1.41	**0.74**	**1.01**	**0.74**	0.76	0.44	0.98	1.15	**0.95**	0.71	**0.16**	**0.08**
DeePHF@ωB97M-V(G&T)	1.26	1.01	1.71	1.27	**0.40**	0.31	1.38	1.12	1.00	0.85	0.78	0.21

Table S6: The performance of different models on other subsets of GMTKN55 is evaluated in terms of MAE (kcal/mol), considering only neutral, closed-shell systems containing C, H, O, and N elements.

Subsets	No. of data	MN15-L	M06-2X	ωB97X-D3	MN15	ωB97X-V	ωB97M-V	XYGJ-OS	XYG3	DeePHF@HF	DeePHF@PBE	DeePHF@B3LYP	DeePHF@M06-2X	DeePHF@ωB97M-V
		Basic properties and reaction energies for small systems												
G2RC	12	8.33	2.30	4.70	3.14	4.26	2.14	2.69	3.13	0.89	0.86	0.96	0.95	0.92
FH51	44	3.41	1.46	2.46	1.94	2.92	1.65	1.57	1.35	1.12	0.52	0.55	0.34	0.37
TAUT15	13	0.69	0.51	1.06	0.97	0.99	0.35	1.17	0.74	0.39	0.39	0.38	0.43	0.32
DC13	10	8.58	7.38	6.33	6.66	7.69	5.38	3.31	4.62	6.05	4.74	4.34	3.90	3.65
DARC	14	2.47	2.04	1.27	1.09	4.82	1.15	0.94	1.78	0.90	0.84	0.82	0.61	0.53
		Reaction energies for large systems and isomerization reactions												
BSR36	36	3.19	4.37	4.85	0.54	2.30	0.31	2.54	1.21	1.20	0.57	0.75	0.69	0.39
CDIE20	20	1.96	0.57	0.70	0.73	0.62	0.57	0.23	0.50	0.44	0.53	0.26	0.25	0.23
ISO34	34	2.20	1.42	1.17	1.67	1.38	0.77	0.71	1.02	0.46	0.36	0.34	0.29	0.28
ISOL24	16	3.64	3.15	3.00	2.57	4.15	2.40	1.72	2.36	2.28	2.67	2.63	1.29	1.11
		Reaction barrier heights												
BH76	5	2.35	1.03	0.88	1.92	1.01	1.55	1.39	0.68	2.79	1.10	0.65	0.69	0.31
BHPERI	22	1.91	1.60	2.65	1.43	1.99	1.17	2.17	0.49	1.65	1.07	0.61	0.60	0.74
BHDIV10	5	2.79	1.11	1.04	1.11	1.31	1.67	1.54	1.68	1.41	0.77	0.57	0.81	0.71
INV24	13	1.05	1.27	1.16	2.30	1.06	1.17	0.72	0.54	2.27	2.74	1.68	2.86	2.62
BHROT27	19	0.84	0.39	0.37	0.46	0.29	0.25	0.32	0.34	0.56	0.43	0.29	0.20	0.15
PX13	8	4.66	6.21	2.60	2.59	3.36	3.20	2.65	1.45	6.89	6.82	6.76	4.83	4.64
WCPT18	12	1.78	2.51	2.45	2.45	2.78	2.90	1.79	1.66	1.38	2.08	1.36	0.98	1.22
		Intermolecular noncovalent interactions												
ADIM6	6	3.17	0.45	0.41	1.50	0.15	0.17	1.19	1.22	2.24	1.13	2.28	0.85	1.53
S22	22	1.58	0.49	0.35	0.70	0.32	0.40	0.64	0.44	2.75	1.67	2.14	0.43	1.50
S66	66	1.36	0.36	0.52	0.63	0.35	0.45	0.66	0.58	2.12	1.02	1.61	0.39	1.13
WATER27	15	2.77	14.04	15.38	11.48	14.98	16.10	14.43	13.08	12.41	2.90	2.90	1.56	7.76
CARBHB12	4	0.61	0.54	0.89	0.39	0.65	0.54	0.41	0.42	0.66	0.22	0.42	0.52	0.32
		Intramolecular noncovalent interactions												
IDISP	6	7.89	1.83	2.84	4.11	2.57	1.76	2.22	1.86	2.53	2.82	2.64	0.78	1.28
ICONF	4	0.69	0.38	0.39	0.59	0.35	0.26	0.13	0.30	1.23	0.91	0.29	0.35	0.24
ACONF	15	0.88	0.35	0.06	0.70	0.04	0.10	0.22	0.24	0.16	0.06	0.08	0.08	0.09
Amino20x4	72	0.87	0.33	0.26	0.55	0.22	0.22	0.13	0.12	1.03	0.75	0.54	0.53	0.31
PCONF21	18	3.92	1.07	0.33	1.43	0.59	0.92	0.47	0.26	2.61	1.07	1.46	1.23	1.70
MCONF	51	1.30	0.46	0.45	0.69	0.39	0.46	0.14	0.09	1.06	1.03	1.13	0.43	0.65
SCONF	17	0.67	0.34	0.28	0.55	0.32	0.58	0.15	0.26	0.58	0.29	0.23	0.28	0.22
BUT14DIOL	64	0.57	0.38	0.41	0.28	0.39	0.38	0.32	0.35	0.20	0.16	0.08	0.09	0.07
Total	643	2.05	1.53	1.64	1.38	1.61	1.19	1.21	1.07	1.61	1.02	1.03	**0.65**	0.89
